# Are parents of intellectually gifted child(ren) at higher, lower or equal risk for parental burnout?

**DOI:** 10.3389/fpsyt.2022.1003167

**Published:** 2022-11-17

**Authors:** Zoé Saliez, Anthony Vandeuren, Isabelle Roskam, Moïra Mikolajczak

**Affiliations:** Institute of Research in Psychological Sciences (IPSY), Université Catholique de Louvain, Ottignies-Louvain-la-Neuve, Belgium

**Keywords:** parent, burnout, wellbeing, parental burnout, antecedents, gifted, high potential, quantitative

## Abstract

Being a parent can lead to exhaustion when risk factors offset protective factors. Recent research enabled the understanding of parental burnout antecedents among parents of typical and atypical children, but we know few about parental burnout (PB) among parents of intellectually gifted (IG) children. At the same time, several qualitative studies report particularities of being a parent of IG child(ren). In this quantitative study, we explore whether the risk of PB is different for parents of IG child(ren) than for the global population. We use two samples of 196 strictly matched parents: the first is composed of parents having at least one IG child, the second is constituted of demographically matched control parents (data collection took place from November 2019 to February 2020). We use Kruskal-Wallis analysis to compare groups. The results suggest that having an IG child does not significantly modify the risk of PB (Mean IG group = 32.45, SD = 28.21; Mean control group = 27.69, SD = 25.58; KW = 3.500, *p* = 0.06; Cohen's d = 0.18). Implications and future perspectives are discussed, including the relevance of taking into account other special features of the IG child and the intellectual giftedness of the parent in future researches.

## Introduction

Intellectual giftedness has been extensively studied. Although different conceptual frameworks exist, many authors define it as a high general intelligence reflected by a high intelligence quotient (IQ) (1, 2). Most of the studies concern the characteristics of intellectually gifted (IG) people: their wellbeing [e.g., (3)], their schooling [e.g., (4, 5)], their sociability [e.g., (6)] etc. Another part of the literature is about how to allow the child to develop his/her potential and satisfy his/her needs [e.g., (7)]. But less is known about the wellbeing of parents of IG children.

Some authors, however, point to the burden that parents of IG children could face. Qualitative studies suggest that parents feel isolated (8, 9). IG children can have difficulties quieting their mind, which is sometimes exhausting for the parent and can complicate aspects of daily life such as bedtime routines (8). IG children may also have emotional overreactions, which require the parent to coach them (8) and which may cause parents to worry about their child's emotional development and adjustment (10). The child can also show an excessive need for attention (8) and a need for constant intellectual stimulation (10). According to Morawska and Sanders (10) parents express doubts about how to manage difficult behavior, concerns about schooling and learning issues and worries about whether the school system is meeting their child's needs. Holland and Pell (11) also point that having children with special educational needs can make parenthood more burdensome. Despite the qualitative evidence, there is, to our knowledge, no quantitative study about whether the intellectual giftedness of the child can influence the wellbeing of his/her parents.

It has recently been shown that when the burden of parenting becomes too heavy, parents are at higher risk for parental burnout (PB) (12). PB manifests itself through four specific symptoms: exhaustion related to the parental role, emotional distancing from one's children, feelings of being fed up with the parental role, and contrast between previous and current parental self (13). PB affects between 0.6 and 8% of parents in Western countries (14). The consequences of this disorder can be severe for the parent (e.g., escape and suicidal ideation) and for their children (e.g., parental neglect and violence) (15, 16). Therefore, understanding risk factors for PB is essential.

PB results from a prolonged imbalance between risk factors (that significantly increase parenting stress) and protective factors (that significantly decrease parenting stress) (12). Although past studies suggest that the child's characteristics weight less than the characteristics of the parent (e.g., emotional intelligence; self-efficacy beliefs) and of the family (e.g., conflict in coparenting), it does not mean that they have no weight. For example, Le Vigouroux and Scola (17) have shown that perceiving one's child as having a high level of neuroticism increases burnout symptoms. In addition, having a chronically ill child or a child with a disability has also been shown to increase the risk for PB [see respectively (18, 19)].

This paper examines the effect of another characteristic of the child on PB: intellectual giftedness. In line with the arguments mentioned at the beginning of the introduction, we may assume that having an IG child would be a risk factor for PB. But we can also think of it as a protective factor: indeed, many IG children achieve better than others at school (20) and have higher emotional intelligence [for a meta-analysis, see (21)], which could make parenting easier.

This research seeks to answer the question “does having at least one IG child modify the risk of PB?” Given that there are arguments for both higher and lower risk, we hypothesize that there would be no difference in PB scores between parents having at least one IG child and control parents, or if there is a difference, that the effect size would be very small.

## Materials and methods

### Participants

Data were collected from a sample of 879 French-speaking parents having at least one child living with them. Among the 879 respondents, 458 parents (52.1%) have at least one IG child (see Procedure below). In order to answer to our research question, parents with IG child(ren) (i.e., having at least an IG child attested by an IQ test realized by a professional) needed to be matched with demographically similar control parents. After the matching procedure (see below), our final sample is composed of 392 parents, including 196 parents of IG child(ren) (later named “IG group”) and 196 control parents (later named “control group”). Table 1 describes sociodemographic characteristics for the IG group and for the control group.

**Table 1:** Sociodemographic characteristics of the sample and equivalence of the control and IG groups.

	**Total**	**Control group**	**IG group**	***p***
	**(*n* = 392)**	**(*n* = 196)**	**(*n* = 196)**	
	***n*** **(%)**			
**Gender**				0.282^a^
Male	34 (8.7%)	20 (10.2%)	14 (7.1%)	
Female	358 (91.3%)	176 (89.8%)	182 (92.2%)	
**Family situation**				1.000^a^
Heterosexual two-parents family	238 (60.7%)	119 (60.6%)	119 (60.6%)	
Single-parent family (separated or divorced)	100 (25.5%)	50 (25.5%)	50 (25.5%)	
Single-parent family (widowed or unmarried)	22 (5.6%)	11 (5.6%)	11 (5.6%)	
Stepfamily	28 (7.1%)	14 (7.1%)	14 (7.1%)	
Homosexual two-parents family	4 (1.0%)	2 (1.0%)	2 (1.0%)	
**Level of education (highest diploma)**				1.000^b^
Primary education	0 (0%)	0 (0%)	0 (0%)	
Middle school	8 (2.0%)	4 (2.0%)	4 (2.0%)	
High school	58 (14.8%)	29 (14.8%)	29 (14.8%)	
Bachelor's degree	128 (32.7%)	64 (32.7%)	64 (32.7%)	
Master's degree	156 (39.8%)	78 (39.8%)	78 (39.8%)	
Post-graduate education	42 (10.7%)	21 (10.7%)	21 (10.7%)	
**Employment status**				0.637^a^
Full-time	167 (42.6%)	81 (41.3%)	86 (43.9%)	
Part-time	153 (39.0%)	81 (41.3%)	72 (36.4%)	
Doesn't work	72 (18.4%)	34 (17.3%)	38 (19.4%)	
**Household net monthly income** ^**d**^				1.000^c^
0 to 1,500€ or 0 to 4,000CHF	28 (7.1%)	14 (7.1%)	14 (7.1%)	
1,500€ to 2,500€ or 4,000CHF to 6,500CHF	80 (20.4%)	40 (20.4%)	40 (20.4%)	
2,500€ to 4,000€ or 6,500CHF to 8,500CHF	118 (30.1%)	59 (30.1%)	59 (30.1%)	
4,000€ to 5,500€ or 8,500CHF to 10,000CHF	80 (20.4%)	40 (20.4%)	40 (20.4%)	
5,500€ to 7,000€ or 10,000CHF to 12,500CHF	46 (11.7%)	23 (11.7%)	23 (11.7%)	
More than 7,000€ or more than 12,500CHF	30 (7.7%)	15 (7.7%)	15 (7.7%)	
Missing values	10 (2.6%)	5 (2.6%)	5 (2.6%)	
**Subjective precariousness**				0.903^a^
Considers oneself as not in precariousness	329 (83.9%)	164 (83.7%)	165 (84.2%)	
Considers oneself as in precariousness	58 (14.8%)	29 (14.8%)	29 (14.8%)	
Considers oneself as in high precariousness	5 (1.3%)	3 (1.5%)	2 (1.0%)	
	**Mean (SD)**			
**Parent's age**	42.92 (7.612)	43.45 (8.993)	42.39 (5.905)	0.159^b^
Missing values	1	1		
**Number of children in the family**	2.13 (0.827)	2.13 (0.828)	2.13 (0.828)	1.000^b^
**Number of children living at home at least 50% of the time**	1.98 (0.806)	1.95 (0.802)	2.01 (0.810)	0.475^b^

a *P*-value was calculated with χ^2^ test.

v *P*-value was calculated with Kruskal-Wallis test for independent samples, as the normality assumption was not respected according to Skewness and Kurtosis indices.

c *P*-value was calculated with t-test as the normality assumption was respected according to Skewness and Kurtosis indices.

^d^Parents were asked to indicate their net monthly income in Swiss francs if they previously indicated that they live in Switzerland. The equivalences were calculated considering the difference in the cost of living in Switzerland compared to Belgium and France.

### Procedure

The present research was approved by the Ethical committee of IPSY (Research Institute in Psychological Sciences of UCLouvain). The research took the form of an online questionnaire available from November 19, 2019 to February 17, 2020 (i.e., before the COVID crisis).

Parents were recruited in the context of a larger study about PB in specific populations [precarious parents, same-sex parents, single parents, parents of teenager(s), parents of disabled child(ren), parents of adopted child(ren) and parents of IG child(ren)]. Control parents were recruited at the same time. Parents of IG children were recruited through three channels: *via* Belgian and French institutions specialized in intellectual giftedness (i.e., Anpeip, HP Repères, CVIM, Mensa, and Singularités Plurielles), through groups and pages specific to intellectual giftedness on social networks and *via* personal acquaintances. Parents were eligible to participate if they had minimum one child living at home at least 50% of the time.

To avoid any possible recruitment bias, the parents were not made aware that the study focused on PB. Instead, it was presented as a study on parental wellbeing and exhaustion. At the beginning of the questionnaire, the parents were informed of the confidential nature of their answers and that they could stop their participation at any time without any justification. After these explanations, they had to consent to participate in the study in order to access the questionnaire itself. By participating, respondents were given the chance to win 200€ (or 220 CHF). Participants had to give their e-mail address if they want to participate to the raffle. They were made aware that the e-mail address was automatically registered in a file separated from the form.

### Measures

#### Socio-demographic factors

Participants were asked their gender, age, country of residence, number of children in the family, and number of children living with them (i.e., children who sleep at home at least half-time). We also asked parents to indicate their children's age and gender, as well as their own educational level, employment status, family situation (e.g., single family, two-parents family, etc.), net monthly household income, and perceived financial situation. Response options for all the variables are available in Table 1.

#### Intellectual giftedness status of the child

Participants were asked to indicate whether they have any IG child(ren) living at home and certified by a professionally conducted IQ test. In Belgium (i.e., the most represented country), a child is considered as IG if he presents an IQ of minimum 125–130. However, we cannot exclude that some professionals demonstrate some flexibility regarding this score, considering the global profile of the child (22).

#### Parental burnout

PB was assessed *via* the Parental Burnout Assessment [PBA; (13)]. The PBA includes 23 items divided into four subscales: exhaustion in one's parental role (e.g., I am so tired of being a parent that I feel sleep is not enough), contrast with previous parental self (e.g., I think I'm no longer the good father/mother I've been for my children), feelings of being fed up with one's parental role (e.g., I cannot stand being a parent anymore) and emotional distancing from one's children (e.g., I do not feel pleasure to be with my children)” (13). Items are rated on a 7-point frequency scale: never (0), a few times a year or less (1), once a month or less (2), a few times a month (3), once a week (4), a few times a week (5), every day (6). The PBA scores range from 0 to 138. Cronbach's alphas are generally good in the present study, both for the whole questionnaire and for each dimension separately (from 0.77 to 0.97).

### Data analysis

We demographically matched the IG group with the control group in order to control for demographic variables in a robust way and be sure that the obtained result is due to the IG status of the child and not to a demographic difference. It has been carried out with the Case control matching tool of Stata 17 (23). For the purpose of knowing on which variables the matching should be done, we had previously examined sociodemographic differences between groups. To do so, we used Chi^2^ tests for categorical variables and Kruskal-Wallis test for ordinal and scale variables since the normality assumption is not respected for the concerned variables. We used Skewness and Kurtosis indices to assess the normality, and we considered the distribution as not normal when the Skewness or the Kurtosis index shows a value of more than twice the value of its standard error in absolute value, as suggested by SPSS website (24). Analyses revealed that four variables were confounding (i.e., Number of children in the family, Household net monthly income, Level of education and Family situation) with α = 0.05. The matching was therefore performed on these variables.

The equivalence of the IG group and the control group after having been matched was checked using Chi^2^ tests for categorical variables, *t*-tests for ordinal and scale variables when the normality assumption was respected and Kruskal-Wallis tests for ordinal and scale variables when the normality assumption was not respected. The normality was assessed by the same way as previously described. Corresponding *p*-values are available in Table 1. The IG group and the control group do not differ on any of the sociodemographic variables after the matching procedure.

To test whether having one or more IG children at home modifies the risk of PB, we first checked the distribution of the PBA. It was not normal according to Skewness and Kurtosis indices. We therefore computed a Kruskal-Wallis test for independent samples. The independent variable was the IG status of children and the dependant variable was the PBA as a continuous variable. All analyses, except the Case control matching, have been computed on SPSS 27 [IBM (25)].

## Results

In the IG group, the mean of the PBA is 32.45 and the standard deviation is 28.21. In the control group, the mean is 27.69 and the standard deviation is 25.58. The Kruskal-Wallis test (index = 3.500; degree of freedom = 1) shows a *p*-value of 0.06 and thus does not display a significant difference between the two groups at the level of α = 0.05. Although the *p*-value is very close to significance, the effect-size remains small (Cohen's d = 0.18).

## Discussion

This research had the objective to assess whether having at least one IG child living at home substantially alters the risk of developing PB. The results indicate that although parents with an IG child have marginally higher PB scores than the control group, the effect size is trivial.

This is in line with results of Mikolajczak et al. (26), showing that particularities of the child play a weaker role in PB development than parent's stable traits, parenting and family-functioning. Moreover, the present findings does not support the idea that the intellectual giftedness of the child would *de facto* burden parenting, as suggested by Guthrie (8), Morawska and Sanders (10) and Wellisch (9). This being said, the very high variance of PB scores in both groups suggests the presence of moderators. We discuss about it in the next section.

### Limitations and potential future topics of research

Despite its strengths, this study has some limitations which may be the starting point of other researches. A first limitation concerns the reduction of our sample in order to create demographically matched groups. The matching procedure made us lose half of our original sample, and one could say that the results may have been different with the whole sample. However, it has likely changed it in a more reliable way because the matching allowed us to control for the impact of demographic variables and therefore increase the robustness of the results.

A second limitation is that the number of IG children in a given family has not been considered, even though having several children with special needs was found to be a risk factor for PB (27).

A third, related, limitation is that we did not ask to parents if their IG child(ren) had another special feature, whereas the study of Gérain and Zech (27) shows that having a child with several special needs is a risk factor for PB. Measuring whether having an IG child with another particularity, such as ADHD, autism or dys-disorders, would be interesting. Indeed, parents of *twice-exceptional* child(ren) (i.e., children with giftedness *and* a disability) experience higher levels of stress (28).

Fourth, we decided to select our samples strictly based on *intellectual* giftedness. Several models of intelligence exist [e.g., (29, 30)], and this complicates any type of study about this topic, as already noted by Carman (31). We used IQ as the selection criterium to make our research more operational, but future studies may consider other criteria or other areas of giftedness.

Fifth, we did not consider parent's giftedness status in this study. It would be very interesting to find out whether there is an interaction effect between the child's and the parent's intellectual giftedness in the emergence of PB. Would the parenthood be easier if the parent and the child have the same IG status, no matter if it is IG or not IG?

Sixth, even though we are glad to see that parents of IG child(ren) are not at higher risk of PB, it does not mean that they do not experience (other) difficulties in their parenthood. For example, they could feel a pressure to allow their child(ren) to develop their full potential. Future studies could explore the experience of IG child(ren)'s parents with other angles of approach.

## Data availability statement

The raw data supporting the conclusions of this article will be made available by the authors, without undue reservation.

## Ethics statement

The studies involving human participants were reviewed and approved by Ethical Committee of IPSY (Research Institute in Psychological Sciences of UCLouvain). The patients/participants provided their written informed consent to participate in this study.

## Author contributions

IR built the online questionnaire and performed the case control matching. AV and ZS collected the data together. ZS performed the other analysis. ZS, AV, and MM have written the manuscript. All authors contributed to the article and approved the submitted version.

## Funding

IR and MM were supported by a Coordinated Research Grant from the French Community of Belgium (ARC Grant No. 19/24–100). This fund did not exert any influence or censorship of any kind on the present work.

## Conflict of interest

The authors declare that the research was conducted in the absence of any commercial or financial relationships that could be construed as a potential conflict of interest.

## Publisher's note

All claims expressed in this article are solely those of the authors and do not necessarily represent those of their affiliated organizations, or those of the publisher, the editors and the reviewers. Any product that may be evaluated in this article, or claim that may be made by its manufacturer, is not guaranteed or endorsed by the publisher.
